# C‐X‐C chemokine receptor CXCR4 mediates diurnal changes in the aggregation and dispersion of CD8 ^+^ T cells within the tumor microenvironment

**DOI:** 10.1002/ijc.70252

**Published:** 2025-11-19

**Authors:** Akito Tsuruta, Marina Fujimoto, Yasuha Hiraoka, Aoi Taniguchi, Yuki Shiiba, Takuto Inoki, Tomoaki Yamauchi, Yuya Yoshida, Naoya Matsunaga, Shigehiro Ohdo, Satoru Koyanagi

**Affiliations:** ^1^ Department of Pharmaceutics Faculty of Pharmaceutical Sciences, Kyushu University Fukuoka Japan; ^2^ Department of Clinical Pharmacokinetics Faculty of Pharmaceutical Sciences, Kyushu University Fukuoka Japan

**Keywords:** circadian rhythm, immune checkpoint inhibitor, tumor microenvironment

## Abstract

Immune checkpoint inhibitors (ICIs) are widely used to treat various types of cancer; however, their effectiveness varies, with some patients exhibiting resistance. Recent studies have shown that the efficacy of ICIs depends on the localization of immune cells, particularly T cells, within the tumor microenvironment (TME). Although the circadian clock is known to regulate immune cell migration into tumors, its role in orchestrating spatially precise intratumoral localization remains unclear. Here, we found that the distribution of CD8^+^ T cells within the TME varied according to the time of day, accompanied by diurnal expression of C‐X‐C chemokine receptor type 4 (CXCR4). The amplitude of the *Cxcr4* expression rhythm was more pronounced in tumor‐infiltrated T cells than in those from the spleen and was associated with time‐dependent changes in their migration toward CXCL12‐expressing cancer‐associated fibroblasts (CAFs). Reanalysis of single‐cell RNA‐seq data from T cells of lung cancer patients also revealed that upregulation of CXCR4 expression in tumor‐infiltrated CD8^+^ T cells was linked to TGF‐β‐SMAD signaling. The TGF‐β‐SMAD signaling‐mediated transactivation of *Cxcr4* was time‐dependently repressed by SMAD7, resulting in diurnal CXCR4 expression. Consequently, administration of a CXCR4 inhibitor during the circadian phase of elevated CXCR4 expression in tumor‐infiltrated CD8^+^ T cells promotes their dispersion throughout tumor tissues, thereby enhancing the efficacy of ICIs. Our findings highlight an unrecognized mechanism underlying diurnal changes in the aggregation and dispersion of CD8^+^ T cells within tumors, offering a novel approach to enhance the anti‐tumor immune effects of ICIs.

## Graphical abstract

What's new?

In addition to governing daily variations in biological activity, the circadian clock regulates immune cell migration into tumors. Whether this influence extends to immune cell localization within the tumor microenvironment (TME) is unknown. Here, using cell and animal models, the authors investigated immune cell distribution within the TME according to time of day. Experiments show that changes in CD8^+^ T‐cell localization are regulated by diurnal CXCR4 expression and TGF‐β–SMAD signaling, allowing for precise spatiotemporal control of tumor immune evasion. Critically, this mechanism influences immune checkpoint inhibitor responsiveness, underscoring the importance of timing in optimizing immunotherapy outcomes.

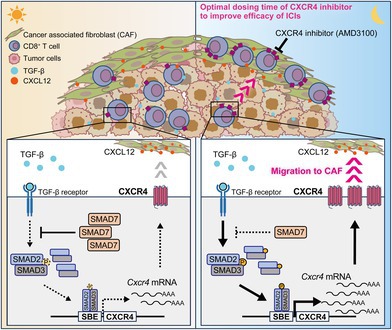

**List of abbreviations**
α‐SMA: alpha‐smooth muscle actin
bp: base pair
CAFs: cancer‐associated fibroblasts
CCR7: C‐C chemokine receptor type 7
cDNA: complementary deoxyribonucleic acid
ChIP: chromatin immunoprecipitation
CXCL12: C‐X‐C motif chemokine ligand 12
CXCR4: C‐X‐C chemokine receptor type 4
DMSO: dimethyl sulfoxide
EDTA: ethylenediaminetetraacetic acid
FACS: fluorescence activated cell sorter
FBS: fetal bovine serum
FCM: flow cytometry
FMO: fluorescence minus one
GATA6: GATA binding protein 6
GEO: gene expression omnibus
GZMB: Granzyme B
GZMK: Granzyme K
HBSS: Hank's balanced salt solution
HOMER: hypergeometric optimization of motif enrichment
ICIs: immune checkpoint inhibitors
IFNγ: Interferon gamma
IL‐2: Interleukin 2
IL7R: Interleukin 7 receptor
i.p.: intraperitoneal
IRF9: Interferon regulatory factor 9
KLF4: Kruppel‐like factor 4
LAG3: lymphocyte activation gene 3
MFI: mean of fluorescence intensity
mRNA: messenger ribonucleic acid
NSCLC: non‐small cell lung cancer
PBS: phosphate‐buffered saline
PCR: polymerase chain reaction
PD‐1: programmed cell death receptor‐1
PDCD1: programmed cell death 1
PD‐L1: programmed cell death‐ligand 1
PEG300: polyethylene glycol 300
RPMI: Roswell Park Memorial Institute
RRID: Research Resource Identification
RT: reverse transcription
SBE: SMAD binding elements
scRNA‐seq: single‐cell RNA sequencing
SELL: Selectin l
SMAD: small mother against decapentaplegic
TAMs: tumor‐associated macrophages
TCF7: transcription factor 7
TGF‐β: transforming growth factor β
TGFBR: TGF‐β receptor
TME: tumor microenvironment
Treg: regulatory T cell
TSS: transcription start site
UMAP: uniform manifold approximation and projection
XCPE1: X gene core promoter element 1
ZT: zeitgeber time

## 1 INTRODUCTION

Immune checkpoint inhibitors (ICIs) have been approved for the treatment of various cancer types, including malignant melanoma, lung cancer, head and neck cancer, and liver cancer, as fourth‐line treatment following radiation therapy, surgical resection, and chemotherapy.^1^, ^2^ ICIs act by targeting immune evasion mechanisms within the tumor microenvironment (TME), which consists of not only cancer cells but also immune cells, fibroblasts, and vascular endothelial cells. Immunosuppressive co‐signaling pathways (immune checkpoints) are mediated by programmed cell death 1 (PD‐1) and its ligand programmed cell death 1 ligand 1 (PD‐L1).^3^, ^4^, ^5^ PD‐1, expressed on CD8^+^ T cells, suppresses cytotoxic activity upon binding to PD‐L1, which is expressed on tumor cells, tumor‐associated macrophages (TAMs), and regulatory T cells (Tregs).^6^, ^7^ Blocking the interaction between PD‐1 and PD‐L1 results in sustained activation of CD8^+^ T cells thereby enhancing their ability to eliminate tumor cells. Consequently, neutralizing antibodies targeting PD‐1 or PD‐L1 have been widely used in cancer therapy. Despite the potential for durable clinical benefits, PD‐1/PD‐L1 blockade therapies achieve positive responses in only a subset of patients (approximately 20%–30%).^8^ Furthermore, acquired resistance to these therapies often leads to tumor progression, even in patients who initially respond favorably.^9^ To address these challenges, numerous studies have been conducted to elucidate the mechanisms underlying ICIs resistance.^9^, ^10^

Daily variations in biological functions are governed by an intrinsic molecular oscillator known as the circadian clock.^11^, ^12^ The circadian clock plays a critical role in regulating both innate and acquired immunity by controlling tissue distribution and immune cell infiltration through cytokine/chemokine‐mediated trafficking and their receptor‐dependent pathways.^13^, ^14^, ^15^, ^16^ Recent molecular and biological analyses have revealed that the circadian clock system is also involved in the regulation of tumor immunity. For instance, the administration of tumor‐antigen‐loaded dendritic cells in the morning has been shown to be more effective in activating T cells.^17^ Additionally, time‐dependent fluctuation of PD‐1‐positive tumor‐associated macrophages (TAMs) was observed in B16/BL6‐formed tumor masses.^18^ In tumor‐bearing mice, T cell infiltration into tumor tissues increases during the dark phase.^19^

A promising therapeutic strategy that exploits these daily variations in immune function to enhance the efficacy of pharmacotherapy or reduce adverse effects is chrono‐pharmacology. This approach involves administering drugs at the time of day when they are most effective and/or best tolerated.^20^ The effectiveness of chrono‐pharmacology has also been demonstrated in ICIs treatments, as evidenced by findings from both experimental animal models and cohort studies involving patients with cancer.^18^, ^19^, ^21^, ^22^ These studies suggest that chrono‐pharmacology could be a valuable approach to overcome immune checkpoint resistance.^18^, ^19^, ^21^, ^22^

Recent spatial analysis of the TME has shown that not only the number of T cells but also their localization within the tumor is critical for the development of ICIs resistance.^10^, ^23^, ^24^ The concept of the tumor immune continuum has been introduced to better characterize the spatial distribution of immune cells. In this framework, tumor immune phenotypes are broadly categorized into three types: [1] the inflamed/infiltrated phenotype, characterized by T cells penetrating the tumor epithelium; [2] the immune‐excluded phenotype, where T cells accumulate in the tumor stroma without entering the tumor epithelium; and [3] the immune‐desert phenotype, marked by a scarcity or complete absence of T cells.^10^, ^23^ Although the infiltration and egression of immune cells are associated with the circadian clock machinery, it remains unclear whether this timekeeping system regulates the precise localization of immune cells within the TME.

During the analysis of intratumoral localization of CD8^+^ T cells, we observed diurnal changes in the aggregation and dispersion of CD8^+^ T cells within tumors formed by mouse Lewis lung carcinoma (LLC1), which were accompanied by diurnal expression of C‐X‐C chemokine receptor type 4 (CXCR4). Temporal elevation of CXCR4 expression on tumor‐infiltrated CD8^+^ T cells promoted their migration toward CXCL12‐expressing cancer‐associated fibroblast (CAFs) high‐density regions, which are distant from cancer cells. Therefore, we investigated whether administration of a CXCR4 inhibitor during the time of day when its expression is elevated on tumor‐infiltrated CD8^+^ T cells causes their dispersion from CAF‐dense regions throughout the tumors, and explored whether resolving intratumoral aggregation of CD8^+^ T cells could enhance the anti‐tumor immune activity of ICIs.

## 2 MATERIALS AND METHODS

### 2.1 Cell and treatment

LL/2 (LLC1) (Research Resource Identifiers [RRID]: CVCL_4538) were obtained from RIKEN BioResource Center (RCB0558). Cells were cultured in Roswell Park Memorial Institute‐1640 medium (RPMI‐1640; Sigma Aldrich, St. Louis, MO) supplemented with 5% Fetal Bovine Serum (FBS; Biowest, Nuaille, France) and 0.25% penicillin–streptomycin (FUJIFILM Wako Pure Chemical, Osaka, Japan). NIH 3T3 (RRID: CVCL_0594) were cultured in Dulbecco's Modified Eagle Medium (DMEM; Thermo Fisher Scientific, Waltham, MA) with 5% FBS (Biowest) and 0.25% penicillin–streptomycin (FUJIFILM Wako Pure Chemical). CTLL‐2 (RRID: CVCL_0227) were obtained from the RIKEN BioResource Center (RCB0637). Cells were cultured in RPMI‐1640 (Sigma Aldrich) supplemented with 10% FBS (Biowest) and 0.25% penicillin–streptomycin (FUJIFILM Wako Pure Chemical) and mouse recombinant IL‐2 (20 ng/mL) (BioLegend, San Diego, CA). To synchronize the circadian clock in CTLL‐2 cells, the cells were incubated in serum‐free media for 2 h, followed by treatment with 50% FBS‐containing media for an additional 2 h. The cells were maintained at 37°C in a humidified 5% CO_2_ atmosphere. All experiments were performed with mycoplasma‐free cells. Cells were confirmed to be free of microbial contamination using a MycoBlue Mycoplasma Detector (Vazyme Biotech Co., Ltd.; Nanjing, China).

### 2.2 Animals and treatments

Male C57BL/6J mice aged 4 weeks were purchased from Jackson Laboratory JAPAN (Yokohama, Japan). To acclimate to the new environment, mice were housed in groups of 6–8 per cage for one week prior to experimentation. They were maintained in a light‐controlled room (zeitgeber time, ZT; ZT0, lights on; ZT12, lights off) at a temperature of 24 ± 1°C and relative humidity of 60% ± 10%, with food and water provided ad libitum. LLC1 (5 × 10^5^ cells) were suspended in 50 μL phosphate‐buffered saline (PBS) and were subcutaneously implanted into the back of C57BL/6J mice under isoflurane anesthesia. After confirmation of tumor engraftment, tumor volume was calculated using the following formula: Tumor volume (mm^3^) = 1/6 × π × long diameter (mm) × short diameter(mm) × height (mm). During the dark period, a dim red light (<5 lx) was used to administer drugs to mice with tumors.

### 2.3 Immunohistochemical staining

LLC1 tumor masses were dissected from tumor‐bearing mice at ZT0, ZT6, ZT12, and ZT18, fixed with 4% paraformaldehyde for 15 min at room temperature, and quenched with PBS containing 250 mmol/L glycine for 30 min. To protect the tissue prior to freezing, the fixed tissues were floated in PBS containing 15% and 30% sucrose. The tissues were embedded in O.C.T. Compound (Sakura Finetek Japan, Tokyo, Japan) and sectioned at 10 μm using CryoStar NX70 (Thermo Fisher Scientific). After blocking with PBS containing 10% FBS and 0.1% Triton‐X for an hour at room temperature, the sections were incubated with anti‐CD8a antibodies (BioLegend, RRID: AB_312740) and anti‐α‐SMA antibodies (BioLegend, RRID: AB_2565041) containing 5% FBS at 4°C for 2 days. The sections were washed twice with PBS containing 0.1% Triton‐X for 15 min and once with PBS for 15 min. After washing, the sections were incubated with secondary antibodies, anti‐mouse Alexa Fluor®488 IgG antibody (RRID: AB_2576208) and anti‐rat Alexa Fluor®555 IgG antibody (RRID: AB_3101870) (Abcam, Cambridge, UK) for 4 h at room temperature. The sections were washed using the same protocol as primary antibodies wash and mounted using VectaShield with DAPI (Vector Laboratories, Newark, CA). Images were visualized using a Zeiss LSM700 (Carl Zeiss, Oberkochen, Germany). The number of CD8^+^ T cells within the CAF‐low density area and high‐density area was counted using a blinded study design.

### 2.4 Fluorescence activated cell sorting analysis

The protocol for the tumor masses and spleens digestion was previously described.^18^ To investigate diurnal variations in tumor‐infiltrated T cells, tumors were dissected at ZT2, ZT6, ZT10, ZT14, ZT18, and ZT22. At each time point, tumors were collected from four individual mice. When tumors were small, two tumors were pooled prior to T cell isolation to ensure sufficient cell yield for sorting. After incubation for 30 min at 37°C, 10% FBS and 12.5 mM EDTA were added to stop the reaction. Dissociated tumor masses and spleens were treated with ACK lysis buffer (BioLegend), and this procedure was repeated twice. Dead cells were stained with the Zombie NIR Fixable Viability Kit or Zombie Violet Fixable Viability Kit (BioLegend). The cells were washed twice with HBSS (−) (Hank's balanced salt solution; 5.4 mmol/L KCl, 440 μmol/L KH_2_PO_4_, 137 mmol/L NaCl, 340 μmol/L Na_2_HPO_4_, 5.6 mmol/L D‐glucose) containing 1% BSA, 25 mmol/L HEPES‐NaOH (pH 7.4), and 2 mmol/L EDTA. To block non‐specific antibody binding by Fc receptors, cells were incubated with Trustain FcX PLUS (anti‐mouse CD16/32 antibody) (BioLegend, RRID: AB_2783138) and True stain Monocyte Blocker (BioLegend) for 10 min on ice. The cells were stained with fluorescence‐labeled antibodies, as listed in Table S1. Data were collected on a BD FACSAria III Cell Sorter or BD FACSAria Fusion Cell Sorter (Becton Dickinson Biosciences, Franklin Lakes, NJ) and analyzed using BD FlowJo (Becton Dickinson Biosciences). The gating strategies for immune cells and CAFs are shown in Figure S1.

### 2.5 Quantitative RT‐PCR analysis

Total RNA was extracted using RNAiso (Takara Bio, Otsu, Japan) or ReliaPrep Miniprep Systems (Promega, Madison, WI) according to the manufacturer's instructions. Complementary DNA (cDNA) was synthesized using a ReverTra Ace qPCR RT Kit (Toyobo LifeScience, Osaka, Japan). The cDNA was amplified by PCR using the THUNDERBIRD SYBR qPCR mix (Toyobo), LightCycler 96 (Roche, Basel, Switzerland), and Applied Biosystems 7500 real‐time PCR system (Thermo Fisher Scientific). The data were normalized using *18S* ribosomal RNA as an internal standard. Primer sequences are listed in Table S2.

### 2.6 Migration assay

CD8^+^ T cells were collected from spleens or tumor masses of LLC1 tumor‐bearing mice at ZT6 and ZT18. Cells were seeded into the upper chamber of a 24‐well Transwell insert (5 μm pore size, Corning, NY) at a density of 2 × 10^4^ cells/mL in 100 μL medium. The lower chamber was filled with 600 μL of T cell culture medium containing 100 ng/mL recombinant mouse CXCL12 (SDF‐1α; BioLegend) or vehicle (PBS containing 0.1% BSA). After incubation for 90 min at 37°C, the number of cells that migrated to the lower chamber was counted using a Cell Titer‐Glo Luminescent Cell Viability Assay (Promega).

### 2.7 Reanalysis of single cell RNA‐seq data

Single‐cell RNA sequencing (scRNA‐seq) data used in this study were obtained from Gene Expression Omnibus (GEO; https://www.ncbi.nlm.nih.gov/geo). The dataset (GSE99254) comprises CD8^+^ T cells collected from the peripheral blood, tumor tissue, and adjacent normal lung tissue of patients with non‐small cell lung cancer (NSCLC). Data visualization and analysis were performed using the Scanpy (v1.8.1) pipeline.^25^ De novo motif analysis was conducted using HOMER with the findMotifs.pl. script.

### 2.8 Preparation of primary CD8 ^+^ T cells

Spleens were harvested from 5‐week‐old male C57BL/6J mice, minced, and passed through a 35 μm mesh filter to obtain single‐cell suspensions. The cells were centrifuged at 300 × g for 5 min at 4°C and resuspended in 1 × Mojosort buffer (PBS containing 0.5% BSA and 2 mmol/L EDTA, pH 7.2). CD8^+^ T cells were isolated using the Mojosort Mouse CD8 T Cell Isolation Kit (BioLegend) following the manufacturer's protocols. The isolated cells were seeded at a density of 1 × 10^6^ cells/mL in six well plates pre‐coated with 3 μg/mL anti‐CD3 antibodies (BioLegend, RRID: AB_312659) and cultured in T cell culture medium. The medium consisted of RPMI supplemented with 10% FBS, 0.5% penicillin–streptomycin, 2 mmol/L L‐glutamine (Sigma Aldrich) and 50 μmol/L 2‐mercaptoethanol (FUJIFILM Wako Pure Chemical) along with 2 μg/mL of anti‐CD28 antibody (BioLegend, RRID: AB_312867). After 3 days of incubation, the cells were used for subsequent experiments.

### 2.9 Construction of reporter and expression vectors

The mouse *Cxcr4* gene 5′‐flanking region spanning from −250 to +100 bp (the number is the distance in base pairs from the putative transcription start site, +1) was inserted into the luciferase reporter vector constructed using Vectorbuilder, *Cxcr4* promoter::Luc. Expression vectors for mouse SMAD7 were constructed using cDNA generated from mouse liver RNA. All coding regions were ligated into the pcDNA3.1(+) vector (Invitrogen) using specific cloning primers containing the restriction enzyme sites listed in Table S3.

### 2.10 Luciferase reporter assay

NIH3T3 cells were seeded at a density of 1.5 × 10^5^ cells/well in 24‐well culture plates. Cells were co‐transfected with 100 ng *Cxcr4* (−250/+100)::Luc and 400 ng vectors expressing SMAD7. pcDNA3.1 was used as an empty vector to ensure equal amounts of DNA across all transfection conditions. Following transfection, cells were stimulated with mouse recombinant TGF‐β1 (10 ng/mL) for 24 h. Cell lysates were prepared and measured using the dual‐luciferase reporter assay system (Promega). The ratio of firefly luciferase activity to Renilla luciferase activity in each sample was used as normalized luciferase activity.

### 2.11 Western blotting

Tumor tissues were collected from LLC1‐bearing mice at ZT6 or ZT18 and lysed with CelLytic MT cell lysis reagent (Sigma‐Aldrich), and centrifuged at 12,000 × g for 10 min at 4°C. The supernatant was collected, mixed with 2 × sample buffer and denatured at 95°C for 5 min. The samples were separated by sodium dodecyl sulfate‐polyacrylamide gel electrophoresis and transferred onto a polyvinylidene difluoride membrane. DynaMarker protein multicolor ladder marker stable II (BioDynamics Laboratory, Tokyo, Japan) was used as a protein ladder marker. The membranes were incubated with antibodies against TGF‐β1 (BioLegend, RRID: AB_10719836), TGF‐β3 (Proteintech, Rosemont, IL; RRID: AB_2202206), α‐SMA (BioLegend, RRID: AB_2565041) or β‐ACTIN conjugated with horseradish peroxidase (Santa Cruz Biotechnology, Santa Cruz, CA, RRID: AB_2714189). The membranes were photographed, and the density of each band was analyzed using ImageQuant LAS4010 (GE Healthcare Life Sciences, Buckinghamshire, UK) and ImageJ software.

### 2.12 Chromatin immunoprecipitation analysis

Mouse primary CD8^+^ T cells (1 × 10^7^) were prepared and incubated with serum‐free medium (0.2% FBS T cell culture medium) for 2 h. The cells were then stimulated with 10 ng/mL TGF‐β1 for 90 min. Subsequently, CD8^+^ T cells were crosslinked with 1% paraformaldehyde for 15 min at room temperature, and the reaction was quenched with 250 mmol/L glycine. The cross‐linked chromatin was sonicated and centrifuged at 13,000 × *g* for 10 min. The obtained supernatant was incubated with 2 μg of anti‐phospho‐Smad3 antibody (Cell Signaling Technology, RRID: AB_2193207) at 4°C overnight. The immunoprecipitated samples were washed and eluted followed by de‐crosslinking via incubation at 65°C for 4 h. To degrade proteins and RNA, the de‐crosslinked samples were incubated with 60 μg/mL Proteinase K and 60 μg/mL RNase A for 1 h. DNA was purified by phenol‐chloroform extraction. The amount of immunoprecipitated DNA was quantified by quantitative PCR analysis.

### 2.13 Drug administration to tumor‐bearing mice

The TGF‐β receptor inhibitor LY364947 (AdooQ Bioscience, Irvine, CA) was intraperitoneally administered (25 mg/kg) to LLC1 tumor‐bearing mice at a dose of 25 mg/kg once daily for three consecutive days at ZT15. LY364947 was dissolved in a solution containing 10% (v/v) DMSO, 40% (v/v) PEG300, and 5% (v/v) Tween80 in saline. Three hours after the final administration, the tumor masses were harvested, and CD8^+^ T cells were isolated by FACS for population analysis. AMD3100 (Tokyo Chemical Industry, Tokyo, Japan) was dissolved in PBS and administered intraperitoneally to LLC1 tumor‐bearing mice at a dose of 10 mg/kg at ZT4 or ZT16, once a day. In the monotherapy of AMD3100, AMD3100 was administered for nine consecutive days. In the combination therapy group, AMD3100 was administered for seven consecutive days. In addition, Anti‐mouse PD‐L1 (B7‐H1)‐InVivo (Selleck, Houston, TX, RRID: AB_3675704) was co‐administered intraperitoneally at a dose of 150 μg/mouse at ZT16, once a day for 5 days, starting from the day after initiation of AMD3100 administration. The mice in the control group received intraperitoneal injections of PBS and IgG2b isotype control‐InVivo (Selleck, RRID: AB_3662740).

### 2.14 Statistical analysis

All statistical analyses were carried out using JMP Pro 17 software (SAS Institute Japan). Results are expressed as mean with SD. All data were checked for normality and equal variances before performing ANOVA. The statistical significance of the diurnal variations was assessed by one‐way ANOVA with Tukey–Kramer post hoc test. The Student's *t*‐test or Welch's *t*‐test was used for independent comparison between the two groups. It was considered significant if the *p* value was <.05.

## 3 RESULTS

### 3.1 Time‐dependent changes in the localization of CD8 ^+^ T cells within LLC1 formed tumors

**FIGURE 1:**
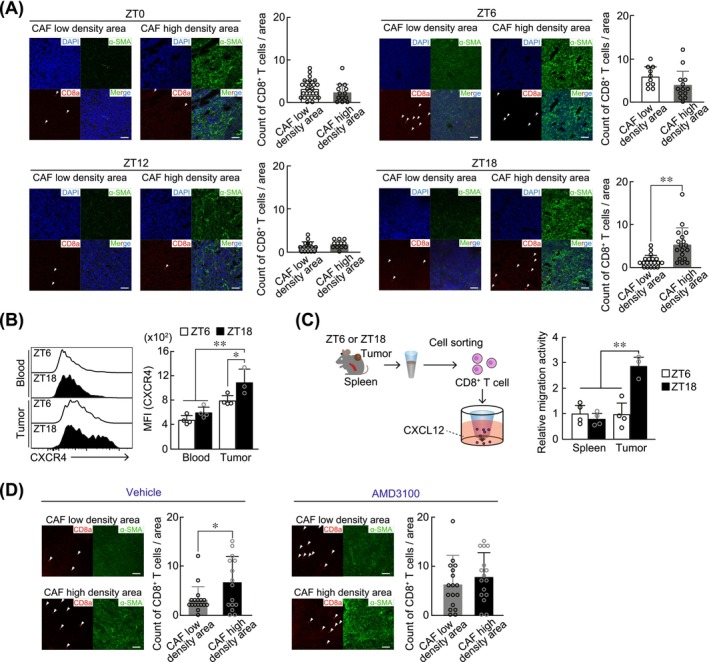
Time‐dependent localization changes in tumor‐infiltrated CD8^+^ T cells in tumor tissue. (A) Time‐dependent differences in the number of tumor‐infiltrated CD8^+^ T cells in the CAFs high‐density area or low‐density area in LLC1 tumor‐masses at ZT0, ZT6, ZT12, and ZT18. Photographs show immunofluorescence staining of α‐SMA as a CAF marker (green) and CD8a as a CD8^+^ T cells marker (red), and nuclear staining with DAPI (blue). The white arrow heads indicate CD8^+^ T cells. Scale bar, 100 μm. Graphs show the numbers of CD8^+^ T cells in CAFs high‐ or low‐density area. The values represent the mean ± S.D. (*n* = 10–26). *p < .05; significant difference between the two groups (*t*_37_ = −1.043 for ZT0, *t*_21_ = −1.555 for ZT6, *t*_25_ = 1.283 for ZT12, *t*_35_ = 3.949 for ZT18, Student's *t*‐test). (B) Time‐dependent changes in the CXCR4 abundance on CD8^+^ T cells prepared from peripheral blood or tumor tissues. CXCR4 expression levels were determined by using flow cytometry (FCM). MFI indicates mean of fluorescence intensity. Values represent the mean ± S.D. (*n* = 3–4). *p < .05; **p < .01; significant difference between the two groups. (*F*_3,10_ = 16.889, *p* < .001; one‐way ANOVA with Tukey Kramer's post hoc test). (C) Time‐dependent differences in CXCL12‐mediated migration of CD8^+^ T cells isolated from spleen or LLC1 tumor‐masses at ZT6 and ZT18. The migration activity of CD8^+^ cells isolated from the spleen at ZT6 was set to 1.0. The values represent the mean ± S.D. (*n* = 3–4). **p < .01; significant difference between the two groups (*F*_5,12_ = 17.430, *p* < .001; one‐way ANOVA with Tukey Kramer's post hoc test). (D) Localization changes in the tumor‐infiltrated CD8^+^ T cells within tumor tissue by intraperitoneal administration of AMD3100 (10 mg/kg) at ZT16 once daily for three days. Left photographs show immunofluorescence staining of α‐SMA as a CAF marker (green) and CD8a as a CD8^+^ T cell marker (red). White arrowheads indicate CD8^+^ T cells. Graphs show the number of CD8^+^ T cells in CAFs in high‐ or low‐density areas. The values represent the mean ± S.D. (*n* = 15–17). *p < .05; significant difference between the two groups (*t*_30_ = 2.199 for vehicle, *t*_31_ = 1.022 for AMD3100; Student's *t*‐test).

To investigate whether intratumoral localization of CD8^+^ T cells varies in a diurnal time‐dependent manner, we performed immunofluorescence staining of tumor samples harvested from LLC1‐bearing mice at ZT0, ZT6, ZT12, and ZT18. Tumor sections were stained with antibodies recognizing α‐SMA, a marker for CAFs and CD8a, a marker for CD8^+^ T cells. The number of CD8^+^ T cells within the CAF low‐density area and high‐density area was counted by blinded observers. During the light phase, CD8^+^ T cells were distributed in both low‐ and high‐density CAF regions; however during the dark phase, these cells tended to localize in the CAF high‐density regions (Figure 1A).

Several chemokines and their receptors play crucial roles in the regulation of T‐cell recirculation.^13^, ^14^, ^15^ Among them, C‐X‐C motif chemokine ligand 12 (CXCL12) and its receptor CXCR4 have been implicated in regulating the intratumoral localization of T cells.^26^ The protein levels of CXCR4 exhibited diurnal variation in tumor‐infiltrated CD8^+^ T cells, but not in circulating CD8^+^ T cells (Figure 1B), with elevated expression observed during the dark phase (ZT18). Analysis of a single cell RNA‐seq database derived from human cancer patients revealed that *CXCL12* was predominantly expressed in CAFs (Figure S2A). Similarly, higher expression of CXCL12 was also observed in CAFs than in myeloids and T cells prepared from LLC1‐formed tumors (Figure S2B). Consequently, we hypothesized that the temporal elevation of CXCR4 expression in tumor‐infiltrated CD8^+^ T cells promotes their migration toward CXCL12‐expressing CAFs. To test this hypothesis, we assessed the CXCL12‐induced migration of CD8^+^ T cells prepared from tumors and spleens at ZT6 and ZT18. Consistent with the diurnal expression pattern of CXCR4 protein on tumor‐infiltrated CD8^+^ T cells, CXCL12‐induced migration of tumor‐infiltrated CD8^+^ T cells was enhanced at ZT18 (*p* < .01), whereas no significant time‐dependent migration activity was observed in CD8^+^ T cells prepared from the spleen (Figure 1C). Furthermore, intraperitoneal administration of AMD3100 (10 mg/kg), a CXCR4 inhibitor, to LLC1 tumor‐bearing mice resulted in the dispersal of CD8^+^ T cells from CAF‐dense regions throughout the tumors (Figure 1D). These data suggest that the diurnal changes in CXCR4 expression on tumor‐infiltrated CD8^+^ T cells underlie their time‐dependent aggregation toward CAF‐dense regions.

### 3.2 TGF‐β‐mediated upregulation of CXCR4 plays a crucial role in regulating the aggregation of tumor‐infiltrated CD8 ^+^ T cells toward CAF‐dense regions

**FIGURE 2:**
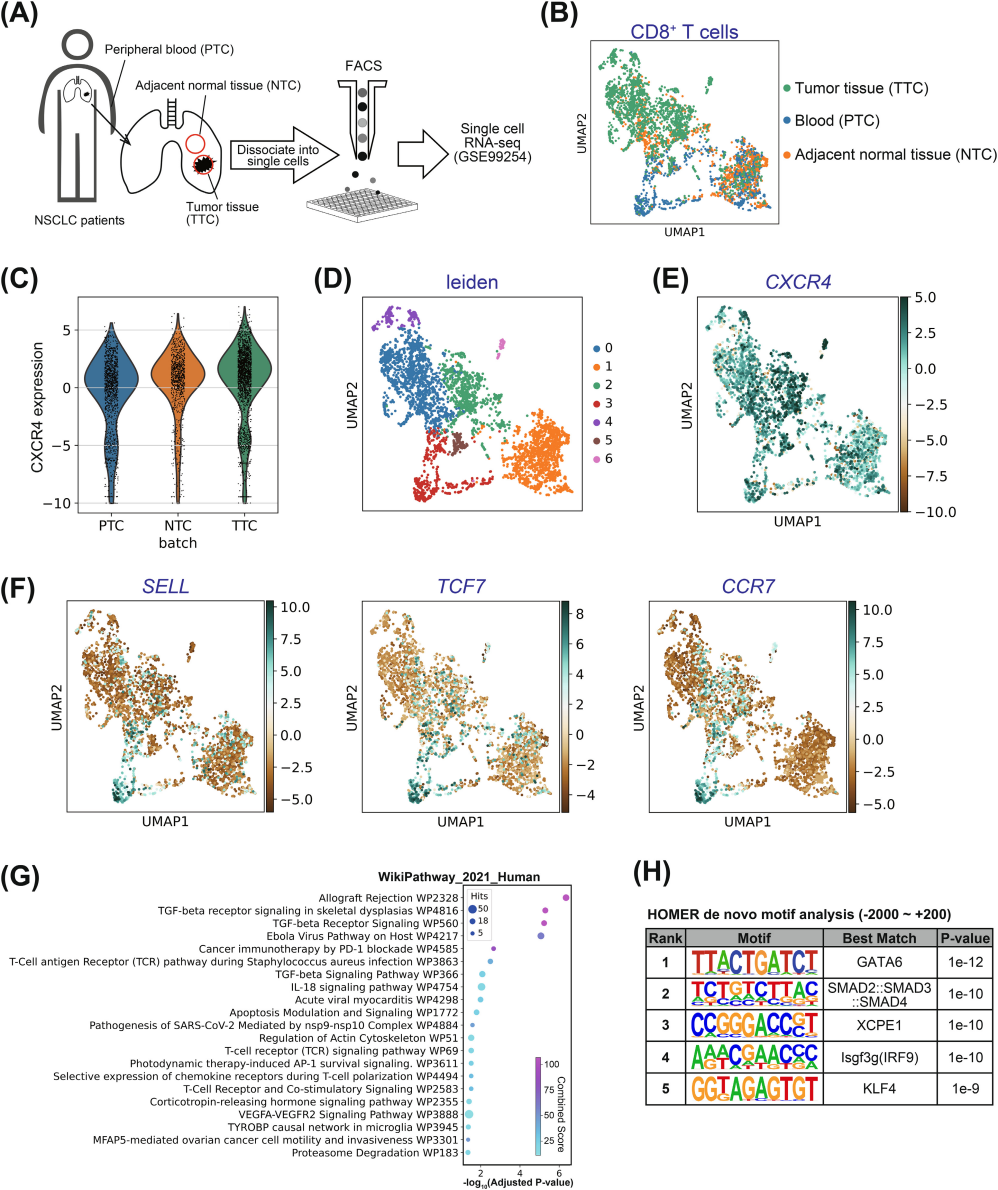
Single‐cell RNA sequence reanalysis of CD8^+^ T cells collected from non‐small cell lung cancer patients (GSE99254). (A) A schematic diagram of CD8^+^ T cell isolation from NSCLC patient tissue. PTC, NTC, and TTC indicate CD8^+^ T cells derived from peripheral blood, adjacent normal tissue, and tumor tissue, respectively. (B) Uniform Manifold Approximation and Projection (UMAP) of the GSE99254 dataset colored according to the tissue of origin of CD8^+^ T cells. (C) Violin plots of *CXCR4* expression in CD8^+^ T cells collected from indicated tissues. (D) UMAP of the GSE99254 dataset colored according to cluster analysis. (E and F) Uniform Manifold Approximation and Projection (UMAP) of the GSE99254 dataset, color‐coded by *CXCR4* expression levels (E) and naive T cell markers *SELL*, *TCF7*, and *CCR7* expression levels (F). (G) Enrichment pathway analysis of the enriched genes in Leiden cluster 2 compared to Leiden cluster 3. The horizontal axis shows the adjusted *p*‐value and the size of the dots represents the number of genes. The colors of the dots indicate the combined scores. (H) HOMER de novo motif analysis of the promoter region from −2000 to +200 bp of the enriched genes in Leiden cluster 2 compared to Leiden cluster 3 using hypergeometric optimization of motif enrichment (HOMER). The motifs were ranked by the *p*‐value with the best match transcription factor.

While LLC1‐bearing mice is widely used as a murine model of lung cancer and provides valuable insights into immune responses and the tumor microenvironment,^27^ there are molecular and immunological differences between LLC1‐bearing mice and human lung cancer. To validate findings from the murine model in a lung cancer patient, we analyzed publicly available single‐cell RNA sequencing (scRNA‐seq) data (GSE99254) from patients with NSCLC.^28^ The use of scRNA‐seq enables high‐resolution characterization of gene expression at the single‐cell level, allowing direct comparison of *Cxcr4* expression patterns across tissue compartments. scRNA‐seq dataset (GSE99254) includes transcriptome profiles of CD8^+^ T cells derived from the peripheral blood, tumor tissues, and adjacent normal lung tissues (Figure 2A). Based on their tissue of origin, CD8^+^ T cells were categorized into three groups (Figure 2B). The expression levels of *CXCR4* mRNA were increased in CD8^+^ T cells collected from tumor tissues (TTC) compared to those from adjacent normal lung tissues (NTC) or peripheral blood (PTC) (Figure 2C). The findings indicate that *CXCR4* expression in tumor‐infiltrated CD8^+^ T cells is upregulated in patients with NSCLC. For further analysis, CD8^+^ T cells were clustered using the Leiden method, identifying cluster 2 as a subgroup with higher *CXCR4* expression (Figure 2D,E). To elucidate the mechanisms driving *CXCR4* upregulation in tumor‐infiltrated CD8^+^ T cells, we performed pathway enrichment and hypergeometric optimization of motif enrichment (HOMER) motif analyses. Pathway enrichment analysis is able to estimate the variable pathways in target cluster compared to control cluster. Furthermore, HOMER motif analysis is able to estimate the activated transcriptional factor in target cluster. By combining these two approaches, we attempted to identify the signaling pathways that are activated in the targeted cluster. These analyses were conducted using highly variable genes in cluster 2 compared to those in cluster 3. Notably, cluster 3 was characterized by elevated expression of naive T cell markers, Selectin L (SELL), Transcription factor 7 (TCF7), and C‐C chemokine receptor type 7 (CCR7) (Figure 2F). Pathway analysis of cluster 2 revealed enrichment of immune‐related terms and TGF‐β signaling pathways (Figure 2G). In addition, HOMER de novo motif analysis identified binding motifs for GATA binding protein 6 (GATA6), SMADs, X gene core promoter element 1 (XCPE1), Interferon regulatory factor 9 (IRF9), and Kruppel‐like factor 4 (KLF4) (Figure 2H). TGF‐β signaling is known to activate SMAD transcription factors through phosphorylation following binding to the TGF‐β receptor.^29^ The consistent identification of TGF‐β‐SMAD signaling terms in both pathway and motif analyses suggested that CD8^+^ T cells in Cluster 2 exhibited upregulated TGF‐β‐SMAD signaling activity.

**FIGURE 3:**
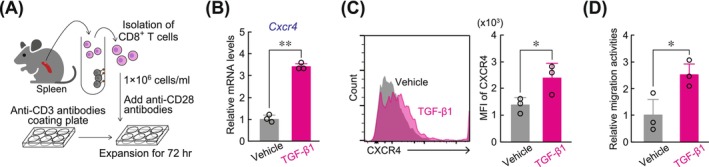
Induction of *Cxcr4* expression in primary CD8^+^ T cells by TGF‐β1 stimulation. (A) Schematic diagram of activation and proliferation method of primary CD8^+^ T cells. (B and C) The expression levels of *Cxcr4* mRNA (B) or CXCR4 protein (C) in CD8^+^ T cells treated with TGF‐β1 (10 ng/mL) or vehicle (PBS containing 1 mg/mL BSA) for 18 h. The mRNA expression levels were normalized by *18S* rRNA levels as internal control, and the value of the vehicle‐treated group was set at 1.0. Values represent the mean ± S.D. (*n* = 3). *p <.05, **p <.01; significant difference between the two groups (*t*_4_ = 17.879 for *Cxcr4* mRNA, *t*_4_ = 2.802 for CXCR4 protein; Student's *t*‐test). (D) CXCL12‐induced migration of CD8^+^ T cells after treatment with TGF‐β1 (10 ng/mL) or vehicle (PBS containing 1 mg/mL BSA) for 24 h. The value of the vehicle‐treated group was set at 1.0. Values represent the mean ± S.D. (*n* = 3). *p <.05; significant difference between the two groups. (*t*_4_ = 3.011; Student's *t*‐test).

**FIGURE 4:**
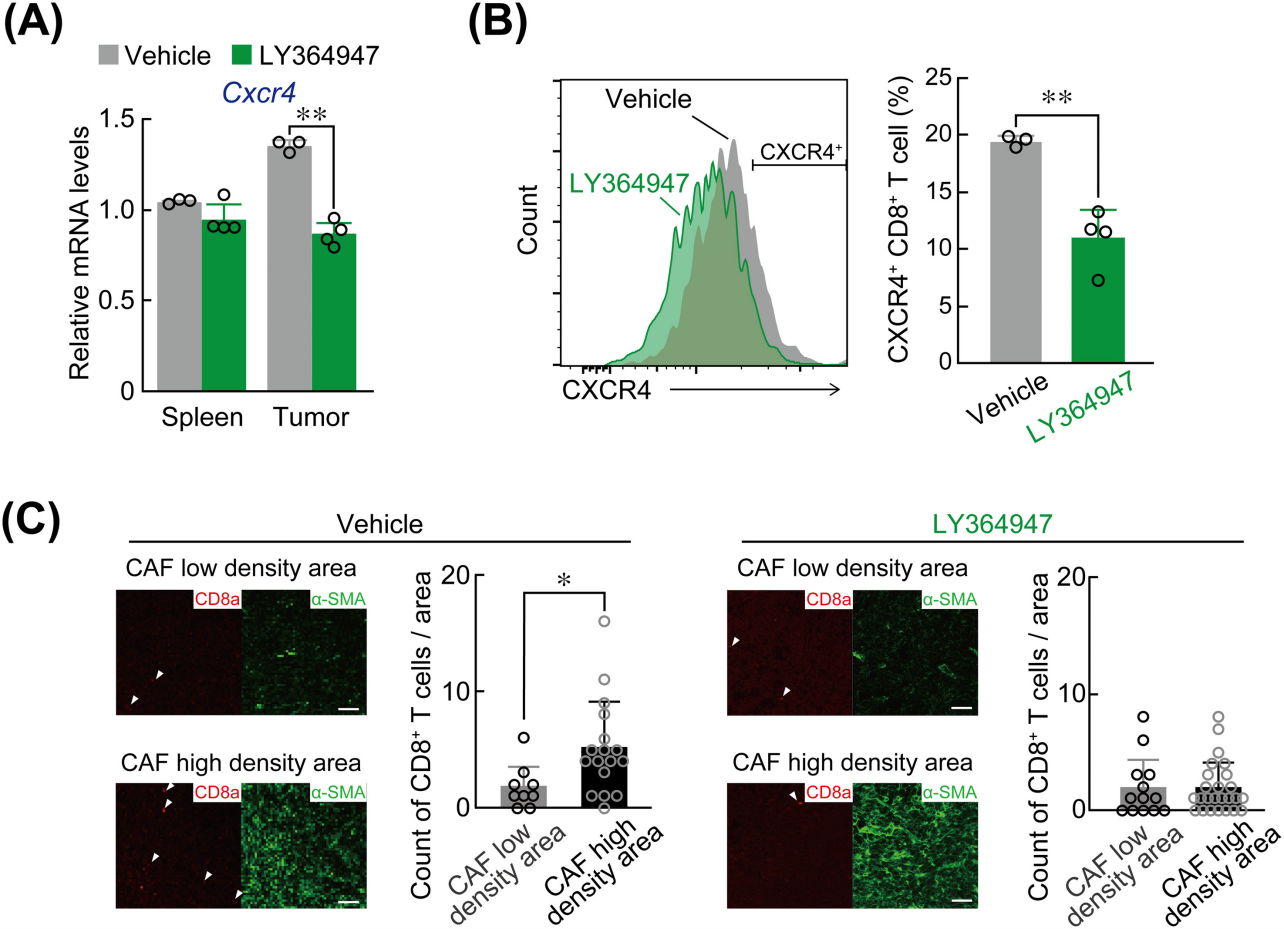
Localization of CD8^+^ T cells to CAF high‐density regions was attenuated by administration of a TGF‐β receptor inhibitor. (A) The levels of *Cxcr4* mRNA in CD8^+^ T cells prepared from spleen or whole LLC1‐formed tumor‐masses. LLC1 tumor‐bearing mice were intraperitoneally (i.p.) administrated with a single daily dose of LY364947 (25 mg/kg), TGF‐β receptor I inhibitor, or vehicle (10 v/v% DMSO, 40 v/v% PEG300, 5 v/v% Tween 80, diluted saline) for 3 days at ZT15. Values represent the mean ± S.D. (*n* = 3–4). **p < .01; significant difference between the two groups (*F*_3,10_ = 36.393, *p <* .001; one‐way ANOVA with Tukey Kramer's post hoc test). (B) The protein levels of CXCR4 in tumor‐infiltrated CD8^+^ T cells prepared from LLC1 tumor‐bearing mice administrated with LY364947 or vehicle. Values represent the mean ± S.D. (*n* = 3–4). **p < .01; significant difference between the two groups (*t*_5_ = 31.139, Student's *t*‐test). (C) Localization changes in the tumor‐infiltrating CD8^+^ T cells within tumor tissue by i.p. administration of LY364947 (25 mg/kg) at ZT15 once daily for 3 days. Photographs show immunofluorescence staining of α‐SMA, as a CAF marker (green) and CD8a as a CD8^+^ T cells marker (red). The white arrow heads indicate CD8^+^ T cells. Scale bar, 100 μm. Graphs show the numbers of CD8^+^ T cells in CAFs high‐ or low‐density area. The values represent the mean ± S.D. (*n* = 9–25). *p < .05; significant difference between the two groups (*t*_23_ = −2.325 for vehicle, *t*_36_ = .004 for LY364947, Student's *t*‐test).

To investigate whether TGF‐β stimulation upregulated *Cxcr4* expression in CD8^+^ T cells, we prepared primary CD8^+^ T cells from mouse spleens (Figure 3A) and treated them with TGF‐β. This treatment significantly increased both *Cxcr4* mRNA and protein levels in the primary CD8^+^ T cells (Figure 3B,C). In addition, TGF‐β1 treatment enhanced CXCL12‐induced migration of primary CD8^+^ T cells (Figure 3D). Intraperitoneal administration of LY364947 (25 mg/kg), a TGF‐β receptor inhibitor, to LLC1 tumor‐bearing mice decreased *Cxcr4* mRNA and protein expression levels in tumor‐infiltrated CD8^+^ T cells (Figure 4A,B). However, this treatment had negligible effects on their expression in CD8^+^ T cells derived from the spleen. Furthermore, the administration of LY364947 to LLC1‐bearing mice during the dark phase led to a redistribution of CD8^+^ T cells, dispersing them from CAF‐dense regions to a more widespread distribution throughout the tumor tissues (Figure 4C). These results suggest that TGF‐β‐mediated upregulation of CXCR4 is involved in the mechanism regulating the aggregation of tumor‐infiltrated CD8^+^ T cells toward high‐density CAF regions.

### 3.3 SMAD3 and SMAD7 function as diurnal regulator of CXCR4 expression

**FIGURE 5:**
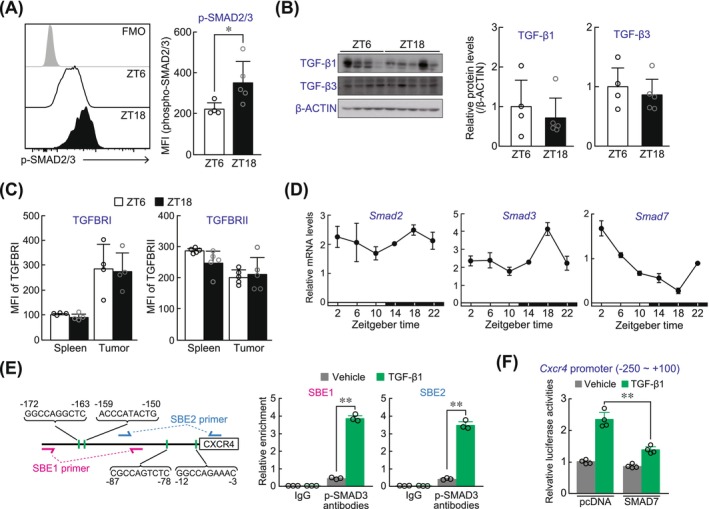
Diurnal oscillation of TGF‐β‐SMAD signaling in tumor‐infiltrated CD8^+^ T cells driven by Smad7. (A) Time‐dependent changes in the phosphorylated SMAD2/3 (p‐SMAD2/3) levels in tumor‐infiltrated CD8^+^ T cells collected from whole tumor tissue. FMO indicates fluorescence minus one. Values represent the mean ± S.D. (*n* = 4–5). *p < .05; significant difference between two groups (*t*_7_ = 2.366; Student's *t*‐test). (B) The protein levels of TGF‐β1 and TGF‐β3 in lysates of tumor tissue collected at ZT6 and ZT18. The left panel shows the photographs of the western blotting. Only one representative blotting photograph of β‐actin is shown. Values represent the mean ± S.D. (*n* = 4–5). (C) The temporal protein expression profiles of TGF‐BRI and TGF‐RII in CD8^+^ T cells prepared from the spleen and whole tumor tissue. Values represent the mean ± S.D. (*n* = 4–5). (D) Diurnal mRNA expression profiles of *Smad2*, *Smad3*, and *Smad7* in tumor‐infiltrated CD8^+^ T cells. The data were normalized to *18S* rRNA levels as internal control. Values represent the mean ± S.D. (*n* = 3). A significant time‐dependent variation was observed. (*F*_5,12_ = 1.725, *p* = .203 for *Smad2, F*_5,12_ = 20.879, *p* < .01 for *Smad3, F*_5,12_ = 108.683, *p* < .01 for *Smad7*; one‐way ANOVA). (E) Binding of phospho‐SMAD3 to SBEs within the 5′‐flanking region of *Cxcr4* gene in primary CD8^+^ T cells treated with vehicle or TGF‐β1. The left panel shows the location of the chromatin immunoprecipitation (ChIP) primer pairs and SBE sites within the 5′‐flanking region from −250 bp to +100 bp of the mouse *Cxcr4* gene. Values represent the mean ± S.D. (*n* = 3). **p < .01; significant difference between two groups (*F*_3,8_ = 1009.035, *p* < .001 for SBE1, *F*_3,8_ = 517.375, *p* < .001 for SBE2; one‐way ANOVA with Tukey Kramer's Post‐hoc test). (F) Luciferase activity derived from *Cxcr4* promoter::Luc in pcDNA3.1‐transfected or SMAD7‐expressing plasmid‐transfected NIH3T3 cells treated with vehicle or TGF‐β1 (10 ng/mL) for 24 h. Data were normalized to the renilla luc activity derived from the *Cxcr4* promoter::Luc plasmids. Values represent the mean ± S.D. (*n* = 4). **p < .01; significant difference between two groups (*F*_3,12_ = 81.865, *p* < .001; one‐way ANOVA with Tukey Kramer's post hoc test).

Upon binding to dimeric ligands, TGF‐β receptor I (TGFBRI) and TGF‐β receptor II (TGFBRII) form heteromeric complexes, inducing conformational changes in the receptor complex that facilitate the direct binding of SMAD2 and SMAD3. This interaction promotes the phosphorylation of SMAD2 and SMAD3 via receptor kinase activity.^30^ In tumor‐infiltrated CD8^+^ T cells prepared from LLC1‐bearing mice, the phosphorylation state of SMAD2/3 exhibited significant time‐dependent variation, with higher phosphorylation levels observed at ZT18 (Figure 5A). Consistent with the previous RNA‐seq data collected from LLC1 cells (Figure S3),^31^ two isoforms of the TGF‐β, TGF‐β1 and TGF‐β3, were detectable in the tumor tissues, whereas TGF‐β2 was not detected. However, their expression levels did not exhibit significant time‐dependent variation in LLC1‐formed tumor tissue (Figure 5B). Furthermore, TGFBRI protein expression, but not TGFBRII expression, was elevated in tumor‐infiltrated CD8^+^ T cells compared to that in splenic CD8^+^ T cells (Figure 5C). In addition, the levels of both receptors did not show significant time‐dependent variations (Figure 5C). These data suggest that time‐dependent changes in CXCR4 expression in tumor‐infiltrated CD8^+^ T cells are not regulated by the expression levels of TGF‐β and its receptors.

Since no time‐dependent variation was observed in TGF‐β signaling stimulating factors, we next focussed on the function of *Smad* genes associated with TGF‐β signaling in tumor‐infiltrated CD8^+^ T cells. SMAD7 attenuates TGF‐β‐induced phosphorylation of SMAD2 and SMAD3.^32^ We therefore examined whether the mRNA expression of *Smad2, Smad3*, and *Smad7* exhibits diurnal variation. While the mRNA levels of *Smad2* did not exhibit a diurnal rhythm, the mRNA levels of *Smad3* and *Smad7* showed significant diurnal oscillations (Figure 5D). The fluctuation in *Smad3* expression closely resembled that of SMAD2/3 phosphorylation (Figure 5A). Furthermore, the diurnal expression of *Smad7* mRNA was nearly in anti‐phase to that of phosphorylated SMAD2/3. These anti‐phasic diurnal changes in SMAD3 and SMAD7 may contribute to the regulation of diurnal variation in *Cxcr4* gene transactivation. Several SMAD‐binding elements (SBEs) were identified in the upstream region of the mouse *Cxcr4* gene by JASPAR database^33^: an open‐access resource for transcription factor binding profiles (Figure 5E left panel). Chromatin immunoprecipitation (ChIP) analysis revealed that TGF‐β1 treatment significantly enhanced the binding of phosphorylated SMAD3 (p‐SMAD3) to these SBEs in primary CD8^+^ T cells (Figure 5E right panel), indicating that this region plays an important role in the upregulation of *Cxcr4* via TGFβ signaling. Furthermore, a region spanning 250 bp upstream and 100 bp downstream of the transcription start site (TSS) of *Cxcr4* gene is cloned into a luciferase reporter vector (*Cxcr4* promoter::Luc), and the transcriptional activity was assessed using a reporter assay. The transcriptional activation of *Cxcr4* promoter::Luc was induced by TGF‐β1 (Figure 5F), which was suppressed by transfection with *Smad7*‐expressing plasmids (Figure 5F). These results suggest that the diurnal expression of *Smad3* and its anti‐phasic expression of *Smad7* regulate the time‐dependent changes in *Cxcr4* levels in tumor‐infiltrated CD8^+^ T cells.

### 3.4 Optimization of dosing schedule of CXCR4 inhibitor enhances anti‐tumor immune effects of ICIs

**FIGURE 6:**
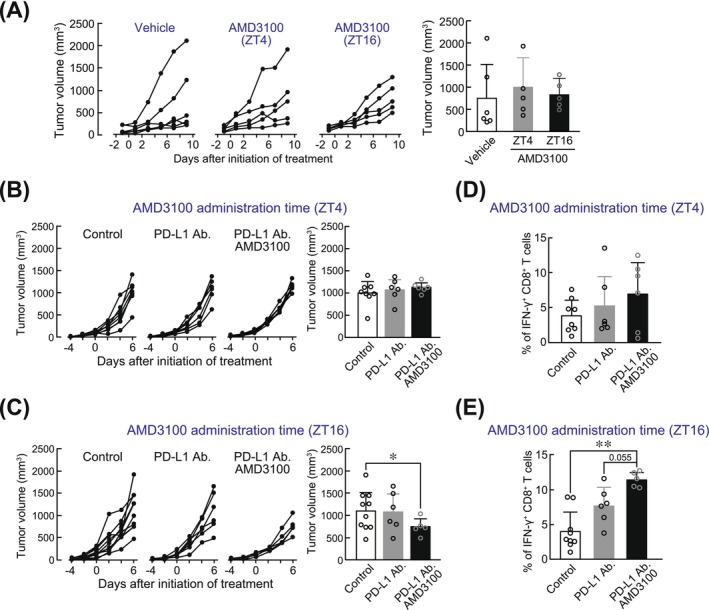
Dosing time‐dependent change in the ability of AMD3100 to activate the CD8^+^ T cells in LLC1 tumor‐bearing mice. (A) LLC1 tumor‐bearing mice were administered AMD3100 once daily for nine consecutive days at ZT4 or ZT16 (*n* = 5–6). The left panel shows tumor growth over time, and the right panel represents tumor volume at 9 days after the initiation of AMD3100 treatment. AMD3100 is a CXCR4 inhibitor. Each value represents the mean ± S.D. (*n* = 5–6). (B and C) LLC1‐bearing mice were concomitantly treated with anti‐PD‐L1 antibodies at ZT16 and AMD3100 at either ZT4 (B; *n* = 6–8) or ZT16 (C; *n* = 5–10). The left panels show tumor growth over time, and the right panels represent tumor volume measured at 6 days after the initiation of treatment. Each value represents the mean ± S.D. (B: *t*_12_ = 1.132 for ZT4 administration; (C) *t*_13_ = 12.965 for ZT16 administration; Welch's *t*‐test). (D and E) The population of IFN‐γ positive CD8^+^ T cells in tumor‐infiltrated CD8^+^ T cells after concomitant treatment with anti‐PD‐L1 antibodies at ZT16 and AMD3100 at either ZT4 (D; *n* = 6–8) or ZT16 (E; *n* = 5–9). Values represent the mean ± S.D. **p < .01; significant difference between the two groups. (D: *F*_2,17_ = 1.009, *p* = .386 for ZT4 administration; E: *F*_2,17_ = 14.900, *p <* .01 for ZT16 administration; one‐way ANOVA with Tukey Kramer's post hoc test).

Since the administration of the CXCR4 inhibitor AMD3100 during the time of day when its expression was elevated in tumor‐infiltrated CD8^+^ T cells facilitated their dispersal from CAF‐dense regions within the tumor microenvironment, we investigated whether monotherapy with AMD3100, as well as combination therapy with AMD3100 and anti‐PD‐L1 antibodies, could improve anti‐tumor immune efficacy. AMD3100 (10 mg/kg) was administered intraperitoneally to LLC1‐bearing mice at ZT4 or ZT16, corresponding to the time points just before the trough and peak of CXCR4 expression in tumor‐infiltrated CD8^+^ T cells, respectively. Tumor growth in LLC1‐bearing mice was not suppressed by AMD3100 monotherapy, regardless of the timing of administration (Figure 6A). In the combination therapy with AMD3100 and anti‐PD‐L1 antibodies, the CXCR4 inhibitor AMD3100 (10 mg/kg) was administered at ZT4 or ZT16, and 150 μg of anti‐PD‐L1 antibodies were administered intraperitoneally at ZT16. This dosing schedule for anti‐PD‐L1 antibodies was based on a previous study indicating that the administration of ICIs during the dark phase is more effective in tumor‐bearing mice.^18^, ^19^ Tumor growth in LLC1‐bearing mice was not suppressed by anti‐PD‐L1 antibody alone compared with control LLC1‐bearing mice (Figure 6B,C). In contrast, co‐administration of AMD3100 and anti‐PD‐L1 antibodies suppressed tumor growth when AMD3100 was administered at ZT16, but not at ZT4 (Figure 6B,C). Furthermore, the protein expression of interferon gamma (IFNγ) was also increased in tumor‐infiltrated CD8^+^ T cells isolated from LLC1‐bearing mice co‐administered with anti‐PD‐L1 antibodies at ZT16 and AMD3100 at ZT16, but not AMD3100 at ZT4 (Figure 6D,E). These results suggest that the administration of a CXCR4 inhibitor can enhance the cytotoxic activity of tumor‐infiltrated CD8^+^ T cells induced by ICIs, particularly when administered at a circadian phase during which CD8^+^ T cells are retained in CAF‐rich regions and segregated from tumor cells within immune‐cold tumor tissues.

## 4 DISCUSSION

Recent spatial analysis of the TME has shown that T cell distribution within the tumor tissue plays a critical role in determining the sensitivity of ICIs.^10^, ^23^, ^24^, ^34^ In the present study, we observed time‐dependent changes in the localization of CD8^+^ T cells within tumor tissue. These changes are regulated by the diurnal expression of CXCR4 in tumor‐infiltrated CD8^+^ T cells, driven by diurnal fluctuations in SMAD2/3‐mediated *Cxcr4* mRNA transcription. Furthermore, combination therapy with anti‐PD‐L1 antibodies and the CXCR4 inhibitor AMD3100 activated CD8^+^ T cells and repressed tumor growth. These data revealed a significant relationship between the diurnal alteration of CXCR4 in tumor‐infiltrated CD8^+^ T cells and the sequestration of CD8^+^ T cells from around the cancer cells. Co‐administration of anti‐PD‐L1 antibodies and CXCR4 inhibitors improved the sensitivity of ICIs in immune‐cold tumors.

The transition from a murine model to lung cancer patient data was methodologically necessary to assess the clinical relevance of our findings. While mouse models provide controlled experimental conditions and mechanistic insights, they do not fully recapitulate the complexity and heterogeneity of human tumor immunology. By incorporating scRNA‐seq data from NSCLC patients, we confirmed that *CXCR4* upregulation in tumor‐infiltrated CD8^+^ T cells is not restricted to experimental systems but is also evident in human disease. This cross‐species validation strengthens the translational impact of our study and underscores the importance of integrative approaches that combine animal models with patient‐derived datasets. Importantly, since mice are nocturnal and humans are diurnal, the circadian rhythm of immune function is often phase‐inverted between the two species.^14^, ^35^ Therefore, it is possible that the diurnal phase of *Cxcr4* expression observed in tumor‐infiltrated CD8^+^ T cells of LLC1‐bearing mice may be opposite to those in humans. This possibility should be carefully considered when extrapolating time‐dependent mechanisms to clinical settings, particularly in the context of chronotherapy or immunomodulation strategies. Future studies are needed to characterize the diurnal dynamics of CXCR4 expression in human CD8^+^ T cells to optimize therapeutic timing and enhance translational relevance.

Under normal conditions, the diurnal expression of *Cxcr4* in CD8^+^ T cells is regulated by glucocorticoid‐driven expression of the interleukin 7 receptor (*IL7R*).^14^ Although glucocorticoids are primarily biosynthesized in the adrenal cortex, a recent study has revealed that myeloids and CD4^+^ T cells also biosynthesize glucocorticoids within the TME.^36^, ^37^ However, the administration of mifepristone, a glucocorticoid receptor inhibitor, did not suppress CXCR4 expression in tumor‐infiltrated CD8^+^ T cells, suggesting that glucocorticoid signaling has a minimal effect on CXCR4 regulation in these cells (Figure S4).

Diurnal expressions of *Smad3* and *Smad7* mRNA were observed in tumor‐infiltrated CD8^+^ T cells. A previous study demonstrated that *Smad3* and *Smad7* expression exhibits circadian rhythms in pancreatic cancer cells and zebrafish,^38^, ^39^ suggesting that their expression may be regulated by intrinsic circadian clock mechanisms. However, in the cytotoxic T cell line CTLL‐2, whose autonomous circadian clock was synchronized by treatment with 50% serum‐containing media for 2 h, no diurnal variation in *Smad7* mRNA expression and only weak diurnal variation in *Smad2* were observed (Figure S5). Notably, *Smad3* mRNA expression in CTLL‐2 cells was extremely low and could not be quantified. These data suggest that the diurnal expression of *Smad3* and *Smad7* in tumor‐infiltrated CD8^+^ T cells may not be regulated by an autonomous circadian clock, but rather by extrinsic signals derived from the tumor microenvironment. Therefore, further detailed analyses using tumor‐infiltrated CD8^+^ T cells are required to elucidate the mechanisms underlying the circadian regulation of *Smad* genes expression within the TME.

A recent analysis of the relationship between tumor immunity and the circadian clock has demonstrated that the efficacy of ICIs is changed by its administration time, with enhanced effectiveness when administered during the active phase (dark phase in mice).^18^, ^19^, ^22^ Dosing‐time‐dependent changes in the efficacy of ICIs are driven by the circadian fluctuation of PD‐1 expression in tumor‐associated macrophages (TAMs) and circadian variation in the infiltration of CD8^+^ T cells.^18^, ^19^ Host anti‐tumor immunity is enhanced during the active phase by increasing the infiltration of CD8^+^ T cells into the tumor tissue, resulting in the enhancement of the efficacy of ICIs in a time‐dependent manner.^19^ On the other hand, our previous study demonstrated that circadian variation in PD‐1 positive tumor‐associated macrophages (TAMs), which are highly expressed during the active phase, contributes to time‐dependent changes in the efficacy of ICIs.^18^ TAMs expressing PD‐1 are known to suppress phagocytic activity and have very low anti‐tumor activity,^40^ suggesting that the activity of anti‐tumor immunity is suppressed during the active phase. Consistent with this observation, the present study also showed that the sequestration of CD8^+^ T cells from the vicinity of tumor cells to the CAF high‐density area within tumor tissue was also enhanced during the active phase due to the rhythmic expression of CXCR4 in CD8^+^ T cells. These data suggest that cancer cells efficiently escape tumor immunity by activating their immune escape mechanisms in conjunction with the timing of activation of the host anti‐tumor immunity.

This study showed that cancer cells escape tumor immunity by localizing CD8^+^ T cells to CAF high‐density regions, which is considered to be similar to the tissue morphology referred to as the excluded phenotype.^41^, ^42^ Excluded phenotypic tumors are classified as immune‐cold tumors and are known to be associated with resistance to immune checkpoint inhibitors. Recent studies have demonstrated that pre‐dysfunctional CD8^+^ T cells, also known as pre‐exhausted or progenitor exhausted CD8^+^ T cells, are enriched in excluded phenotypic tumors.^23^ These cells are also known as a cell population that is activated in response to anti‐PD‐1 antibody therapy, unlike terminally exhausted CD8^+^ T cells, and reactivation of these cells is important for the response to ICI therapy.^43^ Although pre‐dysfunctional cells are characterized by high expression of Granzyme K (GZMK), low expression of Granzyme B (GZMB) and intermediate expression of markers, such as Programmed cell death 1 (PDCD1) and Lymphocyte activation gene 3 (LAG3),^44^, ^45^, ^46^, ^47^ reanalysis of NSCLC scRNA‐seq data (GSE99254) revealed that the *CXCR4* ^high^ CD8^+^ T cell population exhibited *GZMK* ^*high*^ and *GZMB* ^*low*^ expression, suggesting that these cells represent pre‐dysfunctional CD8^+^ T cells (Figure S6). Our present findings on diurnal changes in the localization of CD8^+^ T cells within tumors may be primarily caused by the localization of pre‐dysfunctional CD8^+^ T cells to the CAF region. However, further detailed analysis is required.

TGF‐β promotes the immune‐excluded phenotype by inducing the transition of normal fibroblasts into CAFs and activating these cells.^48^, ^49^ CAFs are also known to produce high levels of TGF‐β, suggesting that CD8^+^ T cells infiltrating CAF high‐density areas may be further stimulated by TGF‐β, thereby accelerating the development of the excluded phenotype. Therefore, combining TGF‐β inhibitors with ICIs has been proposed to enhance the immune‐activating effects of ICIs on excluded phenotype tumors by promoting CD8^+^ T cell infiltration into tumor regions through the elimination of CAFs.^50^, ^51^ In this study, administration of a TGF‐β receptor inhibitor (LY364947) once daily for 3 days dispersed the CD8^+^ T cells from CAF high‐density regions throughout the tumor tissue without reducing α‐SMA, a CAF cell marker, in LLC1 tumor masses (Figure S7). These findings suggest that the TGF‐β receptor inhibitor improves the localization of tumor‐infiltrated CD8^+^ T cells within the tumor both directly by reducing CXCR4 expression on these cells, and indirectly by modulating CAF activity.

The present study demonstrated dynamic diurnal variations in the distribution of CD8^+^ T cells in the TME. Recent research highlights a close relationship between tumor immunity and the circadian clock machinery, with the efficacy of ICIs and the prognosis influenced by dosing time. Additionally, as diurnal rhythm alterations in immune cells are associated with immune evasion mechanisms in cancer cells, optimizing dosing schedules based on the circadian clock dynamics of T cells in the TME may be crucial for effective cancer immunotherapy.

## AUTHOR CONTRIBUTIONS

**Akito Tsuruta:** Conceptualization; data curation; formal analysis; funding acquisition; investigation; methodology; project administration; supervision; visualization; writing – original draft; writing – review and editing; validation. **Marina Fujimoto:** Data curation; formal analysis; methodology; validation; investigation; visualization; writing – original draft. **Yasuha Hiraoka:** Data curation; formal analysis; methodology; investigation; validation; writing – original draft. **Aoi Taniguchi:** Investigation; methodology. **Yuki Shiiba:** Investigation; methodology. **Takuto Inoki:** Investigation. **Tomoaki Yamauchi:** Investigation. **Yuya Yoshida:** Methodology. **Naoya Matsunaga:** Methodology. **Shigehiro Ohdo:** Funding acquisition; project administration; supervision; writing – review and editing. **Satoru Koyanagi:** Writing – review and editing; funding acquisition; project administration; supervision.

## FUNDING INFORMATION

Grant‐in‐Aid for Exploratory Research (22K18375 to S. Koyanagi, 21K18249 to S. Ohdo); Grant‐in‐Aid for Scientific Research A (22H00442 to S. Ohdo); Grant‐in‐Aid for Scientific Research B (25K02425 to S. Koyanagi); Grant‐in‐Aid for Young Scientists (20K16306 and 23K14569 to A. Tsuruta); Grant‐in‐Aid for Scientific Research C (25K10469, to A. Tsuruta). These grants were supported by Japan Society for the Promotion of Science. Platform Project for Supporting Drug Discovery and Life Science Research [Basis for Supporting Innovative Drug Discovery and Life Science Research (BINDS)] from AMED (JP24am121031).

## CONFLICT OF INTEREST STATEMENT

The authors declare no conflicts of interest associated with this manuscript.

## ETHICS STATEMENT

All protocols involving mice were reviewed and approved by the Animal Care and Use Committee of Kyushu University (A22‐022‐0, A24‐114‐0). All procedures were performed in accordance with relevant guidelines and regulations.

## Supporting information


**TABLE S1:** List of antibodies for flowcytometry.
**TABLE S2:** Primer sets for RT‐PCR analysis.
**TABLE S3:** Primer sets for construction of gene expressing plasmids.
**FIGURE S1:** Gating strategies in flowcytometry analysis.
**FIGURE S2:** Identification of CXCL12 expressing cells in lung tumor microenvironment.
**FIGURE S3:** The mRNA expression levels of TGFβ isoforms in LLC1 cells.
**FIGURE S4:** The effect of glucocorticoid receptor inhibitor mifepristone on the CXCR4 expression in CD8^+^ T cells.
**FIGURE S5:** Circadian rhythm of Per2 and Smad7 mRNA expression in CTLL 2 cells synchronized by 50% FBS.
**FIGURE S6:** The expression levels of GZMB and GZMK in CD8^+^ T cells.
**FIGURE S7:** Effect of LY364947 administration on αSMA expression in LLC1 tumor masses.

## Data Availability

Data sources and handling of the publicly available datasets used in this study are described in the Materials and Methods. Further details and other data that support the findings of this study are available from the corresponding authors upon request.
