# Supplementary material for: C‐X‐C chemokine receptor CXCR4 mediates diurnal changes in the aggregation and dispersion of CD8 + T cells within the tumor microenvironment
Source: Int J Cancer. 2025 Nov 19;158(6):1740–54. doi: 10.1002/ijc.70252 (PMC12811207; doi:10.1002/ijc.70252)
Supplement: Supplementary file 1 — TABLE S1: List of antibodies for flowcytometry. TABLE S2: Primer sets for RT‐PCR analysis. TABLE S3: Primer sets for construction of gene expressing plasmids. FIGURE S1: Gating strategies in flowcytometry analysis. FIGURE S2: Identification of CXCL12 expressing cells in lung tumor microenvironment. FIGURE S3: The mRNA expression levels of TGFβ isoforms in LLC1 cells. FIGURE S4: The effect of glucocorticoid receptor inhibitor mifepristone on the CXCR4 expression in CD8+ T cells. FIGURE S5: Circadian rhythm of Per2 and Smad7 mRNA expression in CTLL 2 cells synchronized by 50% FBS. FIGURE S6: The expression levels of GZMB and GZMK in CD8+ T cells. FIGURE S7: Effect of LY364947 administration on αSMA expression in LLC1 tumor masses. [file IJC-158-1740-s001.pdf]

Supplementary Material

C-X-C chemokine receptor CXCR4 mediates diurnal changes in the aggregation and dispersion of CD8$^+$ T cells within tumor microenvironment

Akito Tsuruta, Marina Fujimoto, Yasuha Hiraoka, Aoi Taniguchi, Yuki Shiiba, Takuto Inoki, Yuya Yoshida, Naoya Matsunaga, Shigehiro Ohdo, and Satoru Koyanagi

Table of contents:

Supplementary Table S1 List of antibodies for flowcytometry

Supplementary Table S2. Primer sets for RT-PCR analysis

Supplementary Table S3. Primer sets for construction of gene expressing plasmids

Supplementary Figure S1 Gating strategies in flowcytometry analysis.

Supplementary Figure S2 Identification of CXCL12 expressing cells in lung tumor microenvironment.

Supplementary Figure S3 The mRNA expression levels of TGFβ isoforms in LLC1 cells.

Supplementary Figure S4 The effect of glucocorticoid receptor inhibitor mifepristone on the CXCR4 expression in CD8$^+$ T cells.

Supplementary Figure S5 Circadian rhythm of *Per2* and *Smad7* mRNA expression in CTLL-2 cells synchronized by 50% FBS.

Supplementary Figure S6 The expression levels of GZMB and GZMK in CD8$^+$ T cells.

Supplementary Figure S7 Effect of LY364947 administration on αSMA expression in LLC1 tumor masses.

Supplementary Table S1

Antibodies	Source	RRID
FITC anti-mouse CD3 antibody	Biolgened	AB_312661
PerCP/Cyanine5.5 anti-mouse CD4 antibody	Biolgened	AB_2563023
PE anti-mouse CD8 antibody	Biolegend	AB_312747
APC anti-mouse CD45 antibody	Biolgened	AB_312977
APC anti-mouse/human CD140b antibody	Biolegend	AB_2043971
PerCP anti-mouse/human CD11b antibody	Biolegend	AB_2129375
APC/Fire750 anti-mouse CD8a antibody	Biolegend	AB_2572113
Brilliant Violet 510 anti-mouse CD45	Biolegend	AB_2563061
PE conjugated anti-mouse TGF-bRI antibody	R&D	AB_10890557
PE conjugated anti-mouse TGF-bRII antibody	R&D	AB_2242259
Human/mouse CXCL12 antibody	R&D	AB_2088149
PE anti-mouse CXCR4 antibody	Biolegend	AB_2562783
APC anti-mouse CD11c antibody	Biolegend	AB_313779
APC anti-mouse Ly-6C antibody	Biolegend	AB_1732076
FITC anti-mouse Ly-6G antibody	Biolgened	AB_1236494
PE phospho-SMAD2/3 antibody	Biolegend	AB_11151915

Supplementary Table S2. Primer sets for RT-PCR analysis

Gene symbol	Sequence		Species
Cxcr4	Fw	5'-GACTGGCATAGTCGGCAATG-3'	Mouse
	Rv	5'-AGAGGGGAGTGTGATGACAAA-3'	
Smad7	Fw	5'-GGGCTTTCAGATTCCCAACTT-3'	Mouse
	Rv	5'-AGGGCTCTTGGACACAGTAGA-3'	
Smad2	Fw	5'-ATGTCGTCCATCTTGCCATTC-3'	Mouse
	Rv	5'-AACCGTCCTGTTTTCTTTAGCTT-3'	
Smad3	Fw	5'-CACGCAGAACGTGAACACC-3'	Mouse
	Rv	5'-GGCAGTAGATAACGTGAGGGA-3'	
18s rRNA	Fw	5'-CGGCTACCACATCCAAGGAA-3'	Mouse
	Rv	5'-GCTGGAATTACCGCGGCT-3'	
SBE-160	Fw	5'-CCACGTGGGTAAGGATGGATGC-3'	Mouse
	Rv	5'-ACAGAAGTCCAAGAGCCACTGC-3'	
SBE-12	Fw	5'-CAGTGGCTCTTGGACTTCTGTT-3'	Mouse
	Rv	5'-CCAAACGCCTCAGAGGTTTCA-3'	

Fw; Forward primer, Rv; Reverse primer

Supplementary Table S3. Primer sets for construction of gene expressing plasmids

**Gene name**	**Sequence**		**Species**
Smad7	Fw	5'-ATAGGATCCCGCATGTTCAGGACCAAACG-3'	Mouse
	Rv	5'-ATACTCGAGCTACCGGCTGTTGAAGATGAC-3'	

Fw; Forward primer, Rv; Reverse primer

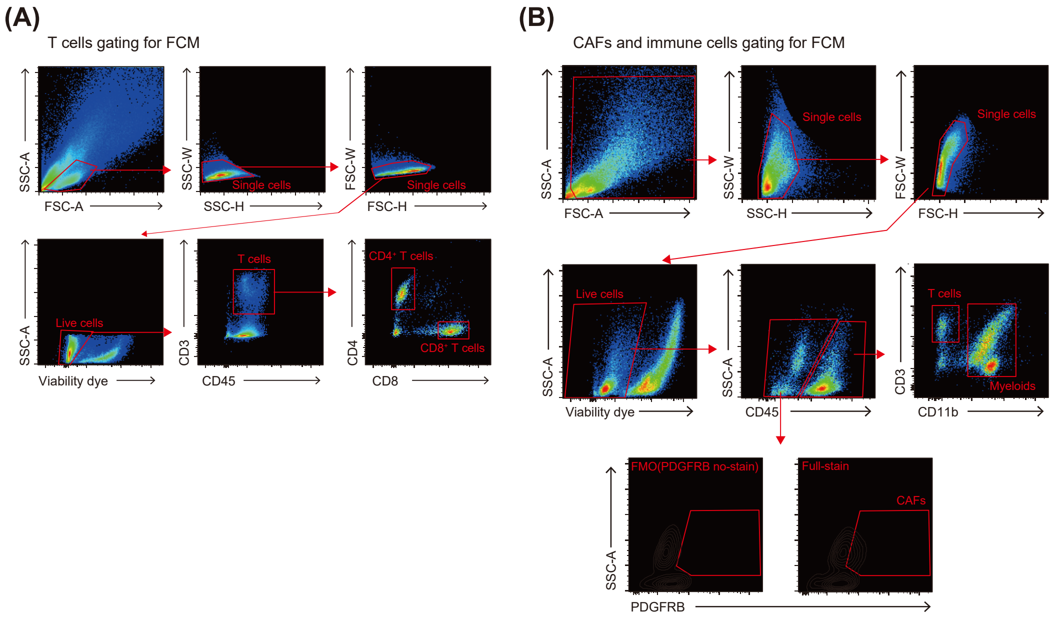

Supplementary Figure S1 Gating strategies in flowcytometry analysis. These strategies were applied to isolate T cells **(A)** and CAFs **(B)** from LLC1-forming tumors implanted in mice.

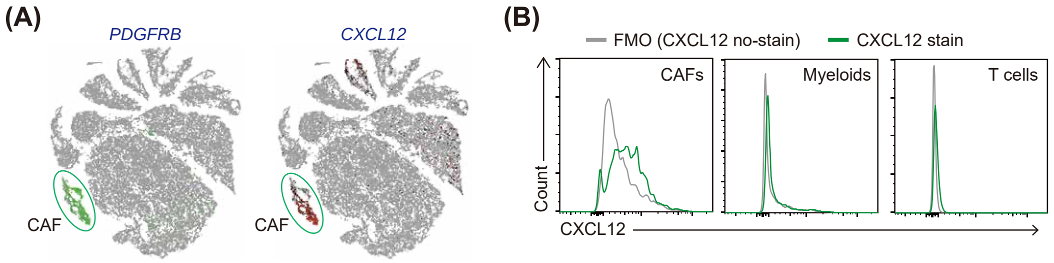

Supplementary Figure S2 Identification of CXCL12 expressing cells in lung tumor microenvironment. A, The analysis of CXCL12 expressing cells in lung tumor dataset from single cell RNA-seq collected from lung tumor patient and visualized with SCope website (**https://scope.aertslab.org/**). PDGFRB is a marker gene of cancer associated fibroblast (CAF). **B,** FCM analysis of CXCL12 expression levels in CAFs, myeloids, and T cells in LLC1-tumor masses dissected from LLC1-tumor bearing mice.

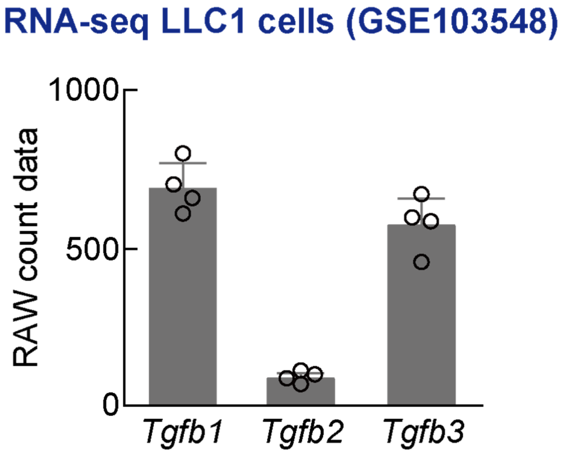

Supplementary Figure S3 The mRNA expression levels of TGFβ isoforms in LLC1 cells. mRNA expression levels of *Tgfb1*, *Tgfb2*, and *Tgfb3* in LLC1 cells were collected from RNA-seq data (GSE103548). Values show the mean with S.D. (n=4).

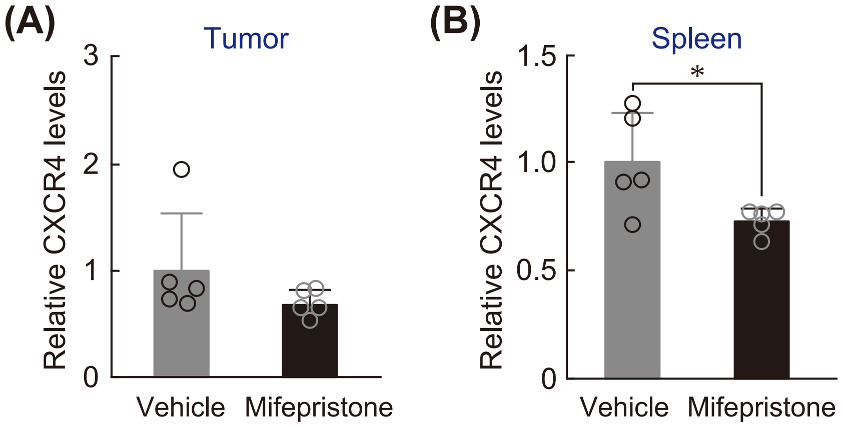

Supplementary Figure S4 The effect of glucocorticoid receptor inhibitor mifepristone on the CXCR4 expression in CD8⁺ T cells. LLC1 bearing mice were intraperitoneally administered mifepristone (60 mg/kg) or vehicle (10% DMSO and 20% β-cyclodextrin) for 3 days once a day at ZT15. **A** and **B,** The expression levels of CXCR4 in tumor-infiltrating $CD8^+$ T cells **(A)** and splenic $CD8^+$ T cells **(B).** The values of vehicle administering mice were set at 1.0. Each value represents the mean with S.D. (n=5). *P<0.05; significant difference between the two groups (t_8=1.314, P=2.225 for tumor-infiltrating $CD8^+$ T cells; t_8=6.678, P=0.032 for splenic $CD8^+$ T cells; unpaired t-test, two sided).

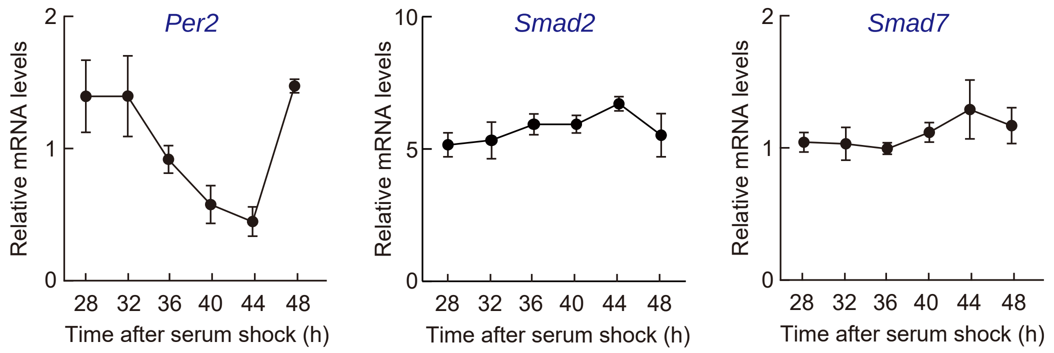

Supplementary Figure S5 Circadian rhythm of *Per2* and *Smad7* mRNA expression in CTLL-2 cells synchronized by 50% FBS. Circadian clock of CTLL-2 cells was synchronized by incubation in media containing 50% FBS for 2 h after incubation in serum free media for 2 h. Values show the mean with S.D. (n=3). ($F_{5,18}$=17.054, P<0.01 for *Per2*, $F_{5,18}$=3.391, P<0.025 for *Smad2*, $F_{5,18}$=2.221, P=0.097 for *Smad7*; one-way ANOVA)

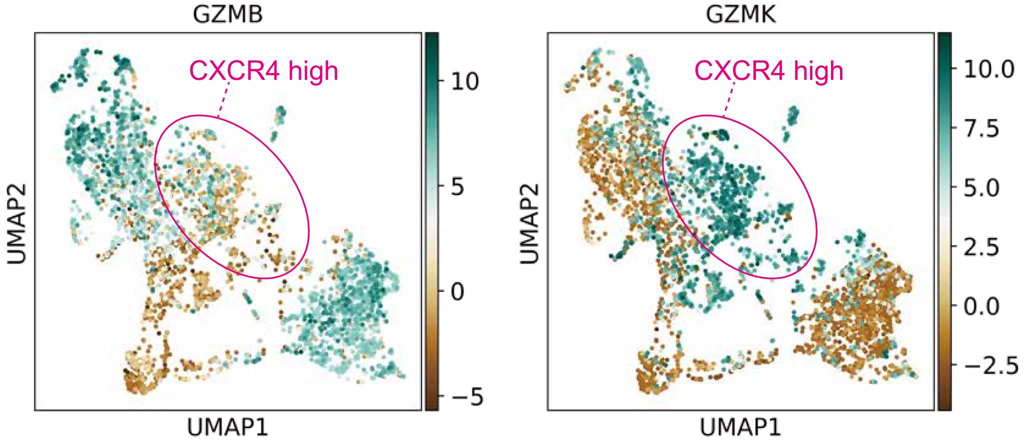

Supplementary Figure S6 The expression levels of GZMB and GZMK in CD8⁺ T cells. UMAP of GSE99254 dataset colored according to GZMB or GZMK expression levels of CD8⁺ T cells derived from NSCLC patients (GSE99254). Green and bronze colors indicate the high and low levels of mRNA expression, respectively.

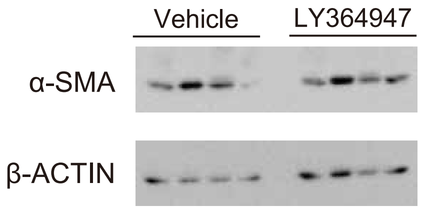

Supplementary Figure S7 Effect of LY364947 administration on αSMA expression in LLC1 tumor masses. LLC1 tumor-bearing mice were intraperitoneally (i.p.) administrated with a single daily dose of LY364947 (25 mg/kg), TGF-β receptor I inhibitor, or vehicle (10 v/v% DMSO, 40 v/v% PEG300, 5 v/v% Tween 80, diluted saline) for 3 days.
